# Non-Covalent Interactions on Polymer-Graphene Nanocomposites and Their Effects on the Electrical Conductivity

**DOI:** 10.3390/polym13111714

**Published:** 2021-05-24

**Authors:** Jorge Luis Apátiga, Roxana Mitzayé del Castillo, Luis Felipe del Castillo, Alipio G. Calles, Raúl Espejel-Morales, José F. Favela, Vicente Compañ

**Affiliations:** 1 Departamento de Física, Facultad de Ciencias, Universidad Nacional Autónoma de México, Circuito Interior s/n, Ciudad Universitaria, Mexico City 04510, Mexico; jl_apatiga@ciencias.unam.mx (J.L.A.); or mitzaye@gmail.com (R.M.d.C.); calles@unam.mx (A.G.C.); espejel@ciencias.unam.mx (R.E.-M.); 2 Departamento de Polímeros, Instituto de Investigaciones en Materiales, Universidad Nacional Autónoma de México, Circuito Interior s/n, Ciudad Universitaria, Mexico City 04510, Mexico; lfelipe@unam.mx; 3 Instituto de Ciencias Nucleares, Universidad Nacional Autónoma de México, Circuito Interior s/n, Ciudad Universitaria, Mexico City 04510, Mexico; francisco.favela@correo.nucleares.unam.mx; 4 Departamento de Termodinámica Aplicada, Escuela Técnica Superior de Ingenieros Industriales (ETSII), Campus de Vera s/n, Universitat Politécnica de Valencia, 46020 Valencia, Spain

**Keywords:** polymer-graphene nanocomposites, conductivity, Monte-Carlo, quantum effects, non-covalent interactions, dispersion correction, polystyrene, polyethylene-terephthalate, polyether-ketone, polypropylene, polyurethane

## Abstract

It is well known that a small number of graphene nanoparticles embedded in polymers enhance the electrical conductivity; the polymer changes from being an insulator to a conductor. The graphene nanoparticles induce several quantum effects, non-covalent interactions, so the percolation threshold is accelerated. We studied five of the most widely used polymers embedded with graphene nanoparticles: polystyrene, polyethylene-terephthalate, polyether-ketone, polypropylene, and polyurethane. The polymers with aromatic rings are affected mainly by the graphene nanoparticles due to the π-π stacking, and the long-range terms of the dispersion corrections are predominant. The polymers with linear structure have a CH-π stacking, and the short-range terms of the dispersion corrections are the important ones. We used the action radius as a measuring tool to quantify the non-covalent interactions. This action radius was the main parameter used in the Monte-Carlo simulation to obtain the conductivity at room temperature (300 K). The action radius was the key tool to describe how the percolation transition works from the fundamental quantum levels and connect the microscopic study with macroscopic properties. In the Monte-Carlo simulation, it was observed that the non-covalent interactions affect the electronic transmission, inducing a higher mean-free path that promotes the efficiency in the transmission.

## 1. Introduction

The science of polymeric materials has revolutionized today’s science and technology. Today, almost every industrial component has a polymer in it [1]; there is a great variety of experimental studies on this topic [2,3,4,5,6,7,8,9]. From this experimental research, it is known that embedding a small number of nanoparticles can change the physical properties of the polymer, e.g., from insulator to conductor [10,11,12]. The most popular fillers that exist are carbon nanotubes [13,14,15], graphene [16], and several oxides [17]. These nanocomposites have a vast spectrum of applications, such as the development of nanoelectronics [18], aerospace industry [19], membrane separation [20], anti-corrosives [21,22], super-capacitors [23,24], or sensors of several interesting molecules as organic molecules [25,26,27]. In particular, carbon nanotubes and graphene nanofillers have proven to drastically increase electrical conductivity. With only a small percentage of particles introduced into the polymer, it is possible to achieve a high conductivity, or even ballistic conductivity, characteristic of pristine graphene [28,29,30].

Experimental and computational studies of conductivity in nanocomposite polymer-graphene present a change in the electrical behavior according to a sigmoid curve [31,32,33]. It is known that the electrical conductivity in some compounds is a sigmoid curve and that the pertinent theory to describe this type of system is to use the percolation theory. The percolation theory is a well-known tool of statistical physics and stochastic processes which has been used often to predict the conductivity of several materials [34,35]. Incorporating the percolation tools and stochastic analysis has led to successful results in terms of modeling electrical conductivity [36,37]. Besides the knowledge of this fact, some details are unknown for polymer-graphene nanocomposites. One of the most interesting facts about the use of carbon nanotubes and graphene nanoparticles as fillers is that the critical point or percolation threshold (PT) occurs with a minimal quantity of nanofillers.

The percolation threshold for carbon nanotubes embedded in polypropylene is around 5% [38,39], whereas, for graphene fillers, the percolation threshold is approximately 18% [31]. It is well known that, to describe the electrical conductivity correctly, it is necessary to consider a proper aspect ratio [31,40].

Our group has proved that quantum effects are the main reason for the percolation threshold occurring so rapidly, and we are developing a methodology to calculate the conductivity of these nano-compounds theoretically. In this work, we simulated the nanocomposites at the microscopic level using an ab-initio computational framework to characterize the quantum effects correctly. Then, we approached the macroscopical level with a Monte-Carlo simulation, which has been proven to give accurate results [41,42,43,44]. Specifically, on the Monte-Carlo simulation, we used the phase-type distribution theory to incorporate the quantum effects in the potential and the conductance estimation. The aspect parameter or action radius was obtained directly from the ab-initio simulation and then incorporated into the Monte-Carlo simulation. To test the methodology and the working hypothesis, we simulated different polymer-graphene nanocomposite membranes: polystyrene graphene (PS-graphene), polyethylene-terephthalate graphene (PET-graphene), polyether-ketone graphene (PEK-graphene), poly-propylene graphene (PP-graphene), and polyurethane graphene (PU-graphene). We compared our outcomes with several experimental results from different authors to validate our model.

## 2. Method

### 2.1. Ab-Initio Calculations

The ab-initio calculations were made to characterize the systems’ quantum effects appropriately and then to incorporate them in the macroscopic simulation and phase conductivity analysis.

**Figure 1:**
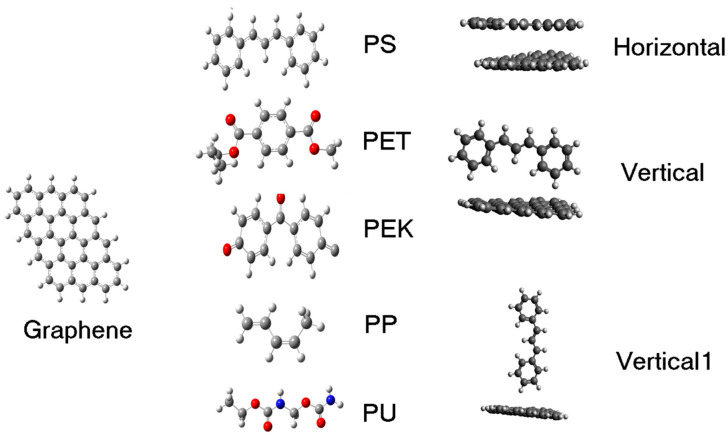
The polymer is placed in several positions over the graphene cluster, and then all the systems are optimized to find the ground-state structure. The systematic search of the stabilized structure started with these configurations: horizontal, vertical, and vertical1 for each polymer with graphene.

The DFT calculations were carried out using the Perdew–Burke–Ernzerhof (PBE) functional [45], Grimme dispersion corrections Bj [46], and 6-311G * (d,p) basis set. We used the computational package Gaussian 16 [47]. The initial structures were placed on a graphene cluster at an initial distance of 2.5 Å. The graphene cluster was designed as a 3 × 4 graphene layer with hydrogen at the edges. The polymer molecules were placed over the graphene sheet in different positions, making a systematic search of the ground-state configurations, see Figure 1. After the adsorption sites and adsorption energies of the ground-state configuration were calculated, we evaluated the deformation to obtain the aspect ratio of the percolation simulation. The adsorption energy was computed with the following equation:

(1) $$E_{ads}=E_{pol+graphene}-\left[E_{pol}+E_{graphene}\right]$$

More details of the ab-initio calculations are presented in the Supporting Information S1. 

### 2.2. Stochastic Simulation

The stochastic simulation is made to emulate the experimental situation as accurately as possible. The simulation is made in three parts: the deposition process of the graphene nanoparticles on the polymer; the accumulation process; and, finally, the electronic transport in the nanocomposite modeling. A summary of the details is:

1. Deposition Process. The random deposition of the graphene nanoparticles over the polymer matrix within a specific percentage is achieved using a Metropolis Monte-Carlo model. The implementation of this model consists of three subparts: a first-neighbors function, the introduction of a training period, and a condition to generate the random deposition of graphene. The details are in the Supporting Information S1;
2. The graphene-clustering Process. It is well known that the deposition process may produce clustered particles over the substrate. In particular, the graphene nanoparticles in a polymer tend to make clusters or fragments that fit together, forming a two-dimensional coverage. In the previous procedure, we could obtain a small concentration of graphene particles or isolate them. Thus, we establish a minimum number of connected graphene particles to be considered a cluster. The detailed algorithm is presented in the Supporting Information S1;
3. Transmission probabilities. The transmission probability is measured using stochastic theory. The transition rate is defined as the probability to pass from the *i*-th to the *j*-th cluster. Then we defined the transition rate matrix or the intensity matrix (*M*) as a function of the Euclidean distance between clusters and each nanocomposite potential. The interaction potential (*u_ij_*) between the *i*-th and the *j*-th clusters was expressed as an expansion of the van der Walls interaction potential proposed by Hamaker [48]. The interaction potential was tested with several terms of different nature (other terms of the power series as ≈ *R*^−6^, *R*^−8^, and *R*^−12^) [49]. In particular, a term of long-range and another of short-range nature [50] form the potential.
  (2) $$u_{i,j}=\frac{1}{dist{\left(i,j\right)}^{6}}I_{dist(i,j)\leq R}+\frac{1}{dist{\left(i,j\right)}^{12}}I_{dist(i,j)>R}$$
  where *dist*(*i*,*j*) is the minimal distance between clusters, the parameter *R* is entirely determined by the nanocomposite and represents the action radius obtained in the ab-initio simulation. The *M* matrix is defined using Equation (2) and is given by the last-passenger model. The inversion of the *M* matrix is almost the Green matrix of the system [50] and allows us to calculate the transition rate. The details are shown in the Supporting Information S1.

In electronic transport theory, the *M* matrix is called the transmission matrix. In particular, in phase-type distribution theory, the *M* matrix is defined *u_i,j_* and *U* = (−*T*)^−1^, where *U* is the expected value in state *j* prior to absorption given initiation in the state *i* [51]. Then, we calculate the value of the heaviest trajectories (i.e., the cumulative probability) in *U*. We repeat this procedure 50,000 times and the output data are the saturation of the simulated polymer. In the Supporting Information S2, an animation of the stochastic simulation is shown.

### 2.3. Experimental Data

To prove our model, we compared the simulation with experimental results, in which the graphene nanoparticles are obtained from graphene oxide nanoparticles and added to the polymer matrix. The simulations made with PS-graphene nanocomposites were compared with the experimental results obtained by Qi et al. [52], in which they measured the conductivity using samples with thickness close to 1 mm at room temperature. For PET-graphene nanocomposites, the comparison was made with the experimental results made by Zhang et al. [53], with a thickness of around 2 mm at room temperature. For PEK-graphene nanocomposites, the experimental measurements were compared with the previous study made by Gaikward and Goyal [54] with samples of ≈ 2 mm of thickness at room temperature, as well. For PP-graphene the experimental results were obtained by a previous work conducted by our group, in which samples had a thickness of 4 mm and the measurements were made at room temperature [31]. For PU-graphene nanocomposites, the electrical conductivity measurements were collected from the experimental work made by Liao et al. [55]. With samples of ≈ 1 mm of thickness and measured at room temperature. To convert the conductance in conductivity, Ohm’s law was used, *G =* *σA*/*L*, in which *A* and *L* are in nanometric dimensions 100 nm^2^ and 10 nm, respectively.

## 3. Results and Discussion

### 3.1. Ab-Initio Results

**Figure 2:**
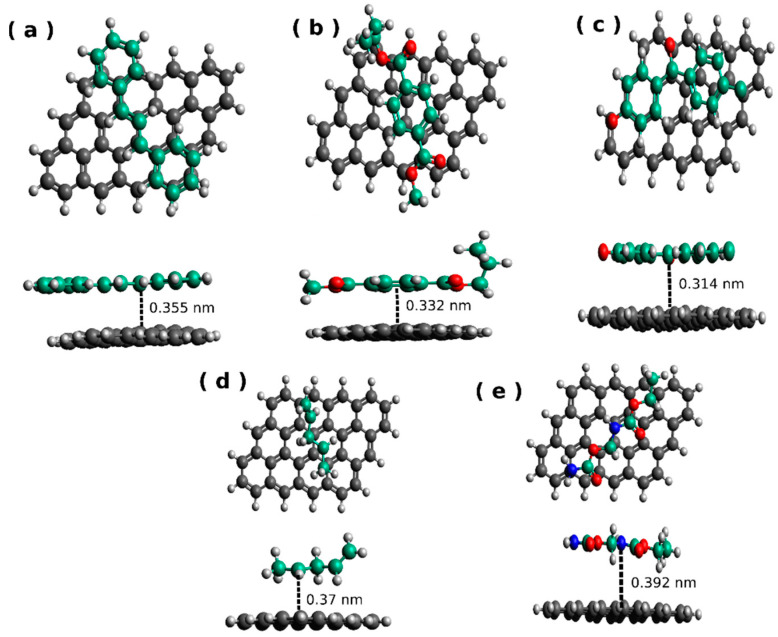
The optimized geometries: (**a**) PS-graphene; (**b**) PET-graphene; (**c**) PEK-graphene; (**d**) PP-graphene; and (**e**) PU-graphene. The carbon of the polymer chain is presented in green with the only purpose of being distinguishable from the graphene cluster.

The systems were optimized to obtain the most stable geometries by performing a systematic search, as was mentioned in the methodology section. These geometries are shown in Figure 2, which locate the polymer parallel to graphene in agreement with experimental evidence [56]. Polymers with aromatic rings (PS-graphene, PET-graphene, and PEK-graphene) settle with the rings of polymer and graphene entirely overlapped, indicating that the C-C is more predominant than C-N or C-O interactions. For polymers with linear configuration (PP-graphene and PU-graphene), the optimized structure shows that the predominant interactions are C-H with the aromatic ring of the graphene. The bond is a non-covalent bond with a π-π stacking interaction for the aromatic polymers and graphene. Meanwhile, for linear polymers, the bond is still in non-covalent type, specifically a CH/π stacking interaction.

The adsorption energies and the HOMO-LUMO gap are shown in Table 1. The polymers with aromatic rings have similar adsorption energies directly consequence the π-π stacking. For linear polymers, such as PP-graphene, and PU-graphene, the adsorption energy, and HOMO-LUMO gap are almost identical. There are mainly two behaviors present here, distinguished by the polymer geometry. The polymers with aromatic rings and π-π stacking and the linear polymers with a CH/π stacking.

A monomer of polystyrene in gas phase presents a gap around 2.37 eV, and polystyrene’s monomers in the presence of graphene have a gap of 0.599 eV. The PET monomer has a HOMO-LUMO gap of 3.58 eV in gas phase and 0.20 eV with graphene interaction. The PEK monomer has a gap of 2.82 eV alone and 0.19 eV with graphene. The linear polymers present the same HOMO-LUMO gap of 0.9 eV representing a huge diminishment from the pristine form of the polymer, which is 3.66 eV for the PP and 5.83 eV for PU. The gas-phase polymers have a big HOMO-LUMO gap in agreement with the insulator properties of the polymer. As soon as graphene nanoparticles are incorporated into the systems, the HOMO-LUMO gap diminished drastically, according to a semiconductor material.

With the optimized structures, adsorption energies, and HOMO-LUMO gap, it was noticed that there is an increasing tendency to improve the electronic transfer when the graphene nanoparticles are introduced in the polymers. The interactions between the graphene and polymers are also weak, forming non-covalent bonds; π-π and CH/π bonds are the predominant interactions presented on the systems.

The direct consequence of the non-covalent bonds is that graphene nanoparticles are stacked parallel to the polymer and enhance the electronic interchange between polymer and graphene.

Following the proposed methodology [31,57], the next step is to analyze the deformation of the system. In elasticity theory, when deformation is induced in a body, the distribution of the molecules is changed, and the body leaves its original state of equilibrium. New forces appear and tend to bring the body to a new equilibrium state with its new molecular configuration. These induced forces are internal stresses and occur due to the interaction forces between molecules or atoms. Internal stresses have a very short action radius. In the traditional macroscopic perspective, the action radius is negligible [58], but it must be quantified in the regimes in which we are working. To quantify precisely, we use the DFT simulation, which tells us precisely how the atomic distribution of the body occurs under the internal stresses due to the non-covalent interactions. 

**Figure 3:**
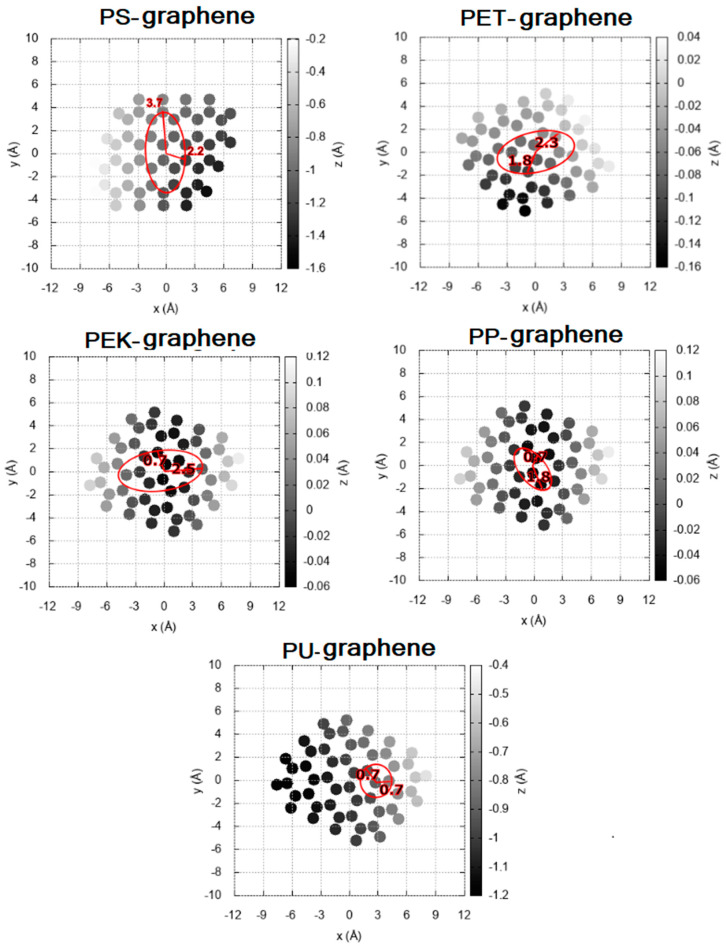
Action radius of the most stable configuration of the systems under study.

The chosen coordinate system has the origin at the center of the graphene surface, and the *z*-axis contains the normal vector of the surface. When the polymer interacts with graphene, carbon atoms can be pushed down (z < 0) or pulled up (z > 0). The action radius is the distance between the maximum and minimum heights near the polymer and directly reflects the internal tensions induced by the polymer–graphene interactions (see Figure 3). The action radius for all the systems are shown in Table 2. 

The deformation on the graphene sheet has an elliptic form. The elliptical shape is acquired due to the way stacking occurs. In order to consider the action radius in the macroscopic simulation, we took the average of the radius of the semi-minor and semi-major axes.

Consequently, the action radius is the direct quantity to measure the induced tensions produced by the non-covalent interactions. The systems with π-π stacking present a more significant deformation due to a higher structural coupling that increases the internal tensions. 

In percolation theory, the aspect ratio is a geometrical parameter representing how close the fillers should be to reach the critical point or the percolation threshold. From our DFT results, the aspect ratio will be the action radius, which is a geometrical parameter that quantifies the internal tensions induced by the non-covalent interactions. The non-covalent interactions are strictly induced by the quantum effects of the electrons of the systems, coming from the dispersion correction [49] and nuclear quantum effects [59,60].

### 3.2. Stochastic Results

The macroscopic calculation was carried out in three parts. The first part is the deposition of graphene nanoparticles on the polymer matrix using a Metropolis Monte-Carlo model. The second part is the formation of graphene clusters over the polymer matrix, incorporating the quantum effects and the internal tensions obtained in the previous section. The third part used a phase-type distribution function to obtain the transmission probabilities (*T*) of electrons. The electronic transport obeys the potential of Equation (2), that consists of some terms of the power series of the dispersion correction. All the results presented here are computed at room temperature (300 K).

In general, the percolation phenomenon can be described by several models as limit-percolation theory or last-passenger percolation theory [36,37]. The limit-percolation theory has gained popularity in recent years as it has been used to describe electronic transport in materials and polymers [36]. However, this model starts from taking an infinite network, where the vertices have an occupation probability given by a Bernoulli distribution, so the results of this model depend only on the shape of the system and not on the material [61]. Several modifications can be made to the limit-percolation theory to consider these aspects. However, these modifications cannot guarantee the correct description of the percolation phenomenon. For example, changing the trajectory of the electrons does not imply percolation in the system [62]. On the other hand, other popular methodologies use the transmission coefficient from the Green function, assuming elastic electrons, periodic systems, zero temperature, and systems with high electrical conductivity. However, we consider that for our systems, these approximations are no longer valid. Therefore, we developed a code that will take these details into account. The purpose of our code was focused on describing in detail the stochastic processes of the systems. We used the phase-type distributions, specifically the percolation of the last passenger, to incorporate the elements of the polymer systems with graphene nanoparticles, such as the inelasticities of the electrons, room temperature, quantum effects, non-covalent interactions, and radii of action. 

Conversely, the last-passenger theory is more flexible than the limit percolation because it applies to finite networks [63] and allows the immediate introduction of the deformation observed in the ab-initio simulation. The nodes are also considered random variables (not Bernoulli probabilities), and allow the inclusion of the properties of the material [56]. The respective interactions between each polymer and the graphene nanoparticles were incorporated in the last-passenger percolation simulation using the potential of Equation (2) and the action radius *R*. With this part of the simulation, it was possible to obtain the transmission probabilities *T* of the electrons transported in the nanocomposites. The Landauer formulation was used to obtain the conductivity of the material [64,65].

Table 3 shows the parameters *R*, the percolation threshold obtained here, and a comparison with experimental studies previously published [31,52,53,54,55]. To compare our results with experiments made by different authors, we convert from vol% or wt% to % of nanoparticles [66] using the graphite bulk density as ≈ 2.2 g·cm^3^ and the molecular weight.

We extrapolate the electrical conductivity (*σ*) as a function of graphene nanoparticles percentage of each simulated set, and we adjust a curve with the sigmoidal Boltzmann equation:

(3) $$\sigma =\sigma_{\max}+\frac{\left(\sigma_{\max}-\sigma_{\min}\right)}{1+e^{(x-PT)/dx}},$$

with *σ*_max_ as the upper limit of the sigmoidal, (*σ*_max_ − *σ*_min_) is the difference between the upper and lower limit, *PT* is the percolation threshold, which represents the critical point in which the conductivity changes drastically, and *dx* represents the slope at the center of the curve. 

**Figure 4:**
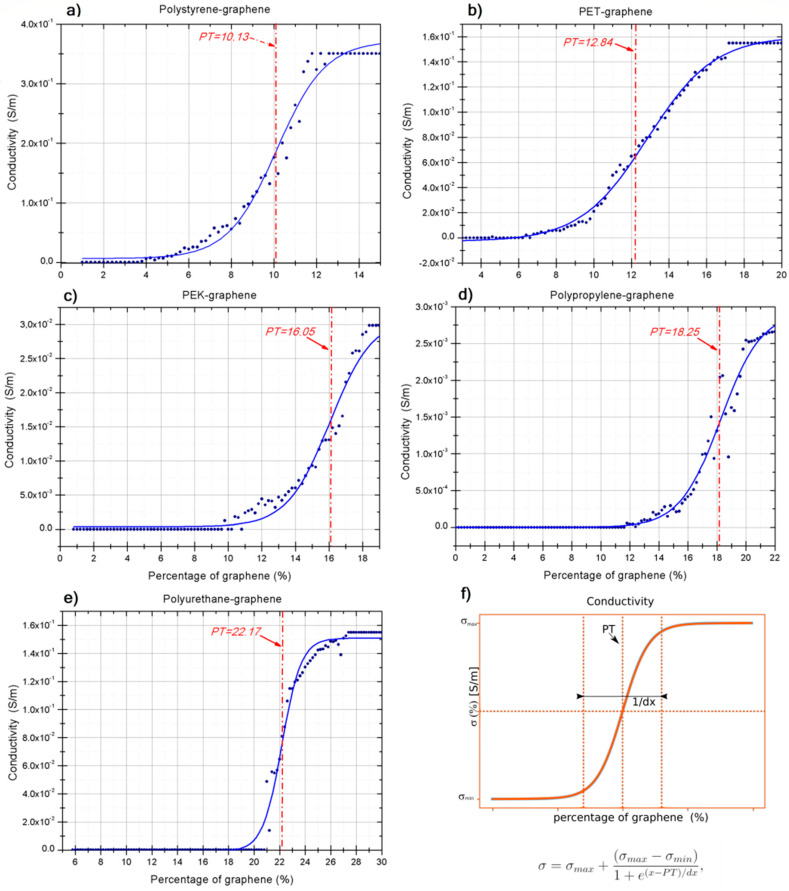
The electrical conductivity versus % of graphene nanoparticles incorporated in the polymer. The blue dots are the values obtained by the simulation, the blue curve is the interpolation made with the sigmoidal Boltzmann, and the vertical red line is the percolation threshold. (**a**) The nanocomposite formed by Polystyrene-graphene, (**b**) PET-graphene, (**c**) PEK-graphene, (**d**) Polypropylene-graphene, (**e**) Polyurethane-graphene. Subfigure (**f**) shows a generalized graphical representation of the sigmoidal Boltzmann to compare with the values obtained in the simulation.

Figure 4 shows the conductivity curve obtained as a function of the percentage of graphene nanoparticles embedded in the polymer matrix. The blue points are the values obtained with our simulation. The blue curve fits the Boltzmann sigmoid curve of our simulation, which follows Equation (3) with the parameters displayed in Table 4. The red vertical line shows the percolation threshold. 

According to Equation (2), we have two terms whose interactional nature presents a subtle difference. The term *R*^−6^ represents the long-range interactions, whilst *R*^−12^ represents the long-range interactions of the non-covalent interactions. These short- and long-range terms directly affect the mean-free path due to the construction of the stochastic simulation. 

The systems in which the long-range terms are predominate are PS-graphene, PET-graphene, and PEK-graphene and have an action radius long enough to transport the electrons. The electronic transfer mechanism acts via the dispersion forces *R*^−6^, indicating that the electrons find a large mean-free path to promote electronic transport, accelerating behavior change from insulating material to a conductor. Therefore, the percolation threshold comes with a percentage much lower than expected. This accelerating is directly a consequence of the π-π stacking and the action radius. 

The systems with dominant short-range terms (*R*^−12^) are PP-graphene and PU-graphene. The action radius is small due to the CH/π stacking, and the nanofillers have a weak interaction with the polymer, so the electrons are transported locally. The mean-free path of the electrons is short, producing low electronic transport compared with other systems studied here. A larger quantity of graphene nanoparticles are introduced to change insulating material to a conductor and improved the trajectories in which electrons can move. As a consequence of this, the percolation threshold occurs with a more significant nanoparticles percentage. 

**Figure 5:**
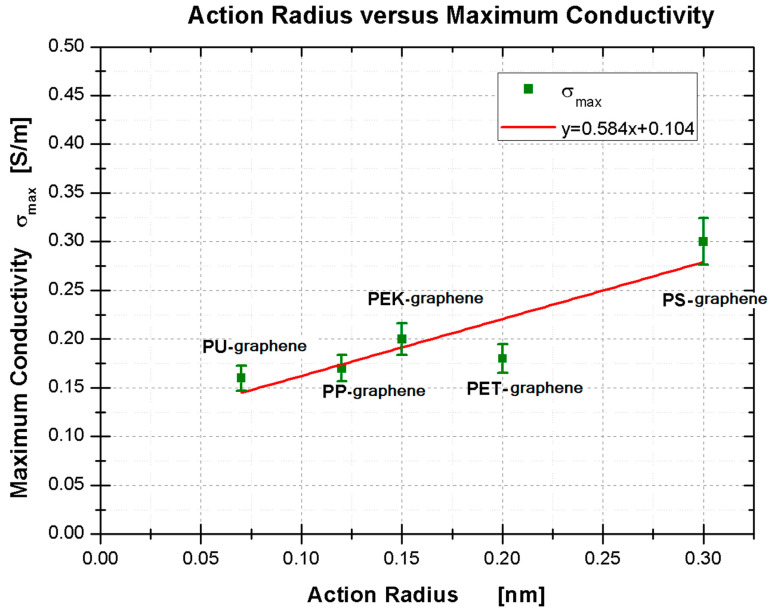
Linear relationship between the action radius versus maximum conductivity.

Additionally, we find a relationship between the action radius and the maximum conductivity. Figure 5 shows the linear relationship between the action radius and conductivity. It displays a tight tendency: polymers with aromatic rings (PS, PET, and PEK) have a higher electrical conductivity than linear polymers (PP and PU). 

## 4. Conclusions

From this study, we prove that quantum effects are crucial to understanding the acceleration of behavior change from insulator to a conductor when graphene nanoparticles are embedded gradually on a polymer matrix. The quantum effects that mainly affect this behavior are the non-covalent interactions. To test our hypothesis, we performed a computational simulation in which graphene nanoparticles are embedded at room temperature in several popular polymer matrices (PS-graphene, PET-graphene, PEK-graphene, PP-graphene, and PU-graphene). We constructed a robust simulation at room temperature with microscopic, macroscopic, and experimental facts to simulate the non-covalent interactions as accurately as possible. 

The non-covalent interactions were characterized in detail with an ab-initio framework and, then, they were included in a Monte-Carlo simulation. The electronic transport is simulated using phase-type distribution, stochastic percolation theory, and Landauer formulation. The electric conductivity of the systems was successfully reproduced in strong agreement with experimental results. We also proved that a stochastic percolation theory with a phase-type distribution is a more appropriate tool because it can incorporate more details than other models, such as considering room temperature or non-covalent interactions. 

Polymers with aromatic rings in their structure are more compatible with graphene nanoparticles to maximize the conductivity, inducing a π-π stacking that increases the transport of electrons from the graphene to the polymer and vice-versa. For polymers with linear structures, the non-covalent interactions are present mainly by the CH/π stacking. Electronic transport is still promoted, although the electrons are not that accelerated compared with the aromatic polymers. It was observed that the non-covalent interactions dominate the electronic transport mechanism and promoted electrons to be transferred efficiently, diminishing the HOMO-LUMO gap. 

The non-covalent interaction increased the deformation of the nanocomposites. The systems that present a more significant deformation have a higher structural coupling; these are the systems that have cyclic rings and higher adsorption energy. We take the aspect ratio as the deformation radius because it characterizes the neighborhood in which the non-covalent interactions are predominant and connects them with the macroscopic properties. The deformation is used on the Monte-Carlo simulation as the aspect ratio, i.e., the action radius *R* of electrons to be transported in the percolation theory. The Monte-Carlo simulation also allows for incorporating the effect of graphene clusterization and considers quantum effects given by graphene–graphene and polymer–graphene interactions. 

For compounds with low percolation thresholds, such as PS, PET, and PEK, the predominant interactions with the graphene nanoparticles are long-range terms, which produces a long mean-free path for the electrons, which are transported efficiently in the nanocomposite. For high percolation thresholds, such as PP and PU, the prevalent interactions are of the short-range type; this means that the effects induced by graphene on the polymer act locally, so the mean-free path of the electrons is shorter than other polymers. It is observed that this model is the most appropriate to describe these systems.

It was proved that the combination of Monte-Carlo simulation with stochastic process based on the phase-type distribution with the last-passenger percolation theory is an excellent approach. As a consequence of the simulation, the electrical conductivity as a function of the percentage of fillers is defined by the Boltzmann sigmoid curve, which allows us to predict the percolation threshold and saturation state correctly at room temperature. The average error of our simulation is around 3%. Specifically, for systems with predominant short-range interactions, our simulation is excellent (≈1% of error). We recognize that interactions of attractive nature contribute significantly to the efficiency of electronic transport by increasing the mean free path of the electrons. The comparison with several experimental studies demonstrates that our model describes the electric transport accurately in polymer-graphene nanocomposites.

## Figures and Tables

**Table 1:** Adsorption energies and HOMO-LUMO gap for the polymer-graphene systems.

System	*E_ads_* (eV)	Gap (eV)
PS-graphene	−0.843	0.60
PET-graphene	−0.880	0.20
PEK-graphene	−0.893	0.19
PP-graphene	−0.509	0.91
PU-graphene	−0.5011	0.90

**Table 2:** Action radius for the polymer–graphene systems.

System	Radius in Semi-Minor Axis (nm)	Radius in Semi-Major Axis (nm)	Action Radius (nm)
PS-graphene	0.22	0.37	0.295
PET-graphene	0.18	0.23	0.205
PEK-graphene	0.07	0.245	0.157
PP-graphene	0.07	0.18	0.125
PU-graphene	0.07	0.07	0.07

**Table 3:** Action radius (*R*), theoretical percolation threshold, experimental percolation threshold, and percentage error. All values are represented as a function of % of graphene nanoparticles.

System	*R* (nm)	PT (% of Graphene)	PT Exp. (% of Graphene)	Error %
PS-graphene	0.3	10.13	10.07 [44]	0.6
PET-graphene	0.2	12.82	11.96 [45]	7
PEK-graphene	0.15	16.05	16.7645 [46]	1.43
PP-graphene	0.12	18.26	18.3 [27]	0.3
PU-graphene	0.07	22.17	22.45 [47]	1.2

**Table 4:** Parameters used in the fitting of the conductivity values, obtained with Equation (3).

System	*σ*_max_ (S/m)	*σ*_max_ − *σ*_min_ (S/m)	PT (%)	dx (%)	〈*R*^2^〉
PS-graphene	0.37	0.36	10.13	1.15	0.986
PET-graphene	0.16	0.1572	12.83	1.78	0.997
PEK-graphene	0.0311	0.0306	16.05	1.27	0.9902
PP-graphene	0.00295	1.0792	18.26	1.42	0.975
PU-graphene	0.1582	0.15002	22.17	0.75	0.994

## Data Availability

The data presented in this study are available on request from the corresponding author.

## Supplementary Materials

The following are available online at https://www.mdpi.com/article/10.3390/polym13111714/s1, the method details are shown in the Supporting Information S1. The Video S2 displays the operation of the stochastic simulation is shown.
