# Supplementary material for: Non-Covalent Interactions on Polymer-Graphene Nanocomposites and Their Effects on the Electrical Conductivity
Source: Polymers (Basel). 2021 May 24;13(11):1714. doi: 10.3390/polym13111714 (PMC8197260; doi:10.3390/polym13111714)
Supplement: Supplementary file 1 [file polymers-13-01714-s001.zip › polymers-1219110-supplementary.pdf]

Supporting Information S1

Non-covalent interactions on polymer-graphene nanocomposites and their effects on the electrical conductivity

Jorge L. Apátiga[1], Roxana M. del Castillo[1], Luis F. del Castillo[2], Alipio G. Calles[1], Raúl Espejel-Morales[1], José F. Favela[3], Vicente Compañ[4,*]

[1] Departamento de Física, Facultad de Ciencias, Universidad Nacional Autónoma de México, Circuito interior s/n, Ciudad Universitaria, Mexico City 04510, Mexico.

[2] Departamento de Polímeros, Instituto de Investigaciones en Materiales, Universidad Nacional Autónoma de México, Circuito interior s/n, Ciudad Universitaria, Mexico City 04510, Mexico.

[3] Instituto de Ciencias Nucleares, Universidad Nacional Autónoma de México, Circuito interior s/n, Ciudad Universitaria, Mexico City 04510, Mexico.

[4] Departamento de Termodinámica Aplicada. Escuela Técnica Superior de Ingenieros Industriales (ETSII). Universitat Politécnica de Valencia, Campus de Vera s/n, 46020-Valencia, Spain

2. Method

2.1 Ab-initio calculations

The geometry optimization of structures, adsorption energies, and action radius were performed with Gaussian 16 software [1]. DFT calculations were made with the generalized gradient approximation (GGA) in the Perdew-Burke-Ernzerhof (PBE) exchange-correlation framework [2]. Due the predominant interactions between the polymer molecules with the graphene are the non-covalents ones, it is necessary incorporated an empirical dispersion correction with the Grimme's method (D3-BJ) [3]. The total energy, with the empirical dispersion is $E_{PBE\text{-}D}=E_{PBE}+E_{disp}$. The empirical dispersion correction is given by $E_{disp}=E^{(2)}+E^{(3)}$. Where $E^{(2)}$ is given by:

$$E^{(2)} = -s_6 \sum_{i=1}^{N_{atoms}-1} \sum_{j=i+1}^{N_{atoms}} \frac{C_6^{ij}}{R_{ij}^6} f(R_{ij})$$

The parameter C_{ij}^{6} is the dispersion coefficient wich represent the quantum interaction between the pair of atoms *ij*, R_{ij} is the distance between those atoms, $s_6=1$ and represents the global scaling factor of the dispersal correction. Then in the D3-BJ is introduced a damping function:

$$f(R_{ij}) = \frac{1}{1+e^{-d(R_{ij}/s_6 R_{ij}{}^{0}-1)}}$$

R_{rj} is the sum of the atomic van der Waals radii of each atom, $d=20$ is the depth of the potential of the damping function, R_{rj}^{0} is the distance at the equilibrium configuration. With the dampinf function, the $E^{(3)}$ energy is:

$$E^{(3)} = \gamma \sum_{ijk} f^{(bj)}(R_{ijk}) \frac{C_9^{ABC}}{(R_{ij}R_{jk}R_{kj})^3},$$

The linear dependent parameter γ depends of the angles between R_{ij}, R_{jk}, and R_{ki}, the coeficient C_9^{ABC} are called the ternary dispersion coefficients and it is obtained by the modified Casimir-Polder integral [4]:

$$C_6^{AB} = \frac{3}{\pi}\int_0^{\infty} d\omega \frac{1}{m}\left[\alpha^{A_m H_n}(i\omega) - \frac{n}{2}\alpha^{H_2}(i\omega)\right] \times \frac{1}{k}\left[\alpha^{B_K H_l}(i\omega) - \frac{l}{2}\alpha^{H_2}(i\omega)\right].$$

Also, the damping function is:

$$f^{(bj)} = s_8 \frac{R_{AB}^{n}}{R_{AB}^{n} + (\alpha_1 R_{0,AB} + \alpha_2)^n},$$

which is finite when $R_{ab} \to 0$, and $s_8=0.7875$ for PBE.

2.2. Stochastic simulation

The stochastic simulation is made to emulate the experimental situation as accurately as possible. The simulation is made in three parts; The deposition process of the graphene nanoparticles on the polymer; The accumulation process, and finally, the electronic transport in the nanocomposite is modeling. All the details of the simulation are explained below.

2.2.1. Deposition Proccess

We used a Metropolis Monte-Carlo model to simulate the random deposition of the graphene nanoparticles over the polymer matrix within a specific percentage of

graphene included in the polymer. The implementation of this model consists of three subparts: a first-neighbours function, the introduction of a training period, and a condition to generate the random deposition of graphene.

The modeling was made over an nxm matrix simulating the polymer. We moved over each cell of the matrix, analyzing its first neighbors; if none of them have graphene nanoparticles, the probability of having (or not) graphene nanoparticles are:

$$P(x_{i,j} \text{ has nanoparticles}) = p,$$

$$P(x_{i,j} \text{ does not has nanoparticles}) = 1 - p.$$

Where p is the probability to find the nanoparticles. If a first neighbour has a graphene nanoparticle, the probability changes as:

$$P(x_{i,j} \text{ has nanoparticles}) = p\ (1+q),$$

$$P(x_{i,j} \text{ does not has nanoparticles}) = 1 - p(1+q).$$

The procedure is repeated until the matrix is completely covered. The parameters p and q depend entirely on the material and are between $0 < p < 1$ and $0 < q < 1/p-1$. The cells at the edge of the matrix have a bias since they have fewer first neighbours than the inner counterparts. To solve this, we introduce a period for training or annealing, which consists of adding as many rows and columns as necessary so that the cells that are on the edges on the original matrix are inner cells in the extended matrix. Once the graphene deposition process is finished, we eliminate the extra rows and columns. In this way, it is forced to have a homogeneous deposition process.

2.2.2. The graphene-clustering process

It is well known that the deposition process may produce clustered particles over the substrate. In particular, the graphene nanoparticles in a polymer tends to make clusters or fragments that fit together, forming a two-dimensional coverage. In the previous procedure, we could get a small concentration of graphene particles or even an isolated one. Thus, we establish a minimum number of connected graphene particles to be considered a cluster.

We set two initial integer values, one to count the number of clusters to be created (*k*), the second value represents the minimal number of elements needed to form a cluster *(N)*. We then proceed to create a new matrix with the coordinates of each cell obtained by the previous step. This new matrix is often called the coordinates matrix *(CM)*.

In general, the algorithm used to produce the cluster is displayed as:

1. It takes an initial element of *CM* (x_0) and evaluates if there is graphene or not in the cell. If it does not have graphene, then it goes on to the next cell. Otherwise, the coordinates of the present cell x_0 will be saved in *CM*.
2. Each time there is a new cell in CM, it evaluates the first neighbors around it and repeat step 1.
3. If the number of elements added to the *CM* is equal or greater than *N*, then it creates a cluster *k* with the elements added in this process and increases the counter *k* by one. Otherwise, it deletes them from the *CM* matrix and sets them as a non-graphene cell in the matrix.
4. Finally, it moves on to the next cell. By the end of this iterative process, we obtain a CM matrix whose elements contain graphene and have been clustered.

2.2.3 Transmission probabilities

We take a sample of *5%* of columns from the beginning and the end of the *CM* matrix. Those clusters have at least one element within the initial limit taken as initial states, defining the initial probability vector *(n)*. These initial states are assigned a uniform probability of an electron starting there. Then, we defined the intensity matrix (M) as a function of the Euclidean distance between clusters and each nanocomposite potential. The interaction potential (u_{ij}) between the *i*-th and the *j*-th clusters was expressed as an expansion of the van der Walls interaction potential proposed by Hamaker [5]. The interaction potential was tested with several terms of different nature (other terms of the power series as ≈ R^{-6}, R^{-8}, and R^{-12}) [6]. In the end, we saw that the systems were described appropriately in terms of quantum nature. In

particular, a term of long-range and another of short-range nature [7] form the potential

$$u_{i,j} = \frac{1}{dist(i,j)^6} I_{dist(i,j) \leq R} + \frac{1}{dist(i,j)^{12}} I_{dist(i,j) > R}, \qquad (2)$$

where *dist(i,j)* is the minimal distance between clusters and the parameter *R* is entirely determined by the nanocomposite and represents the action radius of each term of the power law of the u_{ij}. *I(a≤b)* represents the indicator function and is defined as:

$$I(dist(i,j) \leq R) = \begin{cases} 0, b < dist(i,j) \\ 1, dist(i,j) \leq R \end{cases}$$

We denote the intensity matrix (*M*) as:

$$M(i,j) = \begin{cases} 0, \text{if } \ \mathrm{i} > \mathrm{j} \\ u_{i,j}, \ \text{if } \mathrm{i} < \mathrm{j} \\ -\sum_{i \neq q} M(i,q), \ \text{if } \mathrm{i} = \mathrm{j} \end{cases}$$

It should be noted that *M* is a superior triangular matrix, so the allowed movements are to the right and upwards, which corresponds to the Last-passenger simulation. The absorption state vector *(t)* is defined as the probability of transporting those clusters, which are in the last *5%* of the matrix. The inversion of the submatrix of *M* without the absorption state vectors is the Green matrix of the system [8], which allows us to calculate the transition rate.

In stochastic theory, the transition rate is defined as the probability to pass from the *i*-th to the *j*-th cluster. Meanwhile, in electronic transport theory, the sub-intensity matrix is called the Transmission matrix. In particular, in phase-type distribution theory, the sub-intensity matrix is defined from *M* and $U = (-T)^{-1}$, where *U* is the expected value in state *j* prior to absorption given initiation in state *i* [8]. Now, we calculate the value of the heaviest trajectories (i.e., the cumulative probability) in *U*. We repeat this procedure 50,000 times and the output data is the saturation of the

simulated polymer. In the supplementary material, it is shown how the stochastic simulation occurs.

References

1. Frisch, M. J.; Trucks, G.W.; Schlegel, H.B.; Scuseria,G.E.; Robb,M.A.; Cheeseman, J.R.; Scalmani, G.; Barone, V. ; Mennucci, B. ; Petersson, G.A.; Nakatsuji, H.; Caricato, M.; Li, X.; Hratchian, H.P.; Izmaylov, A.F.; Bloino, J.; Zheng, G.; Sonnenberg, J. L.; Hada,M.; Ehara, M.; Toyota, K.; et al. Gaussian09 Revision E.01, Gaussian Inc. Wallingford CT **2009**.
2. Ernzerhof, M.; Scuseria, G. E. Assessment of the perdew–burke–ernzerhof exchange-correlation functional, The Journal of Chemical Physics **1999** 10,5029–5036. doi:10.1063/1.478401.
3. Grimme, S.; Ehrlich, S.; Goerigk, L. Effect of the damping function in dispersion corrected density functional theory, Journal of Computational Chemistry **2011** 32; 1456–1465. doi:10.1002/jcc.21759.
4. Methfessel, M.; Paxton, A.T.; . High-precision sampling for Brillouin-zone integration in metals. Phys. Rev. B 1989, 40, 3616. DOI: 10.1103/PhysRevB.40.3616.
5. Hamaker, H. The london-van der waals attraction between spherical particles, Physica **1937** 4, 1058-1072. doi:https://doi.org/10.1016/S0031-8914(37)80203-7.
6. Musher, J. I. Calculation of london-van der waals energies, The Journal of Chemical Physics **1963**, 39 2409-574 2417. doi:10.1063/1.575 1734041.
7. Ambrosetti, A.; Ferri, N.; DiStasio, J.; Tkatchenko, A. Wavelike charge density fluctuations and van der waals interactions at the nanoscales, Science **2016** 362, 1171–1176. doi:10.1126/science.aae0509.
8. M. Blat, B. Nielsen, 1 ed., Springer, Germany, **2017**. doi:10.1007/580 978-1-4939-7049-0.
